# Pancreatic Surgery in the Older Population: A Single Institution's Experience over Two Decades

**DOI:** 10.1155/2016/8052175

**Published:** 2016-11-27

**Authors:** Bhaumik Brahmbhatt, Abhishek Bhurwal, Frank J. Lukens, Mauricia A. Buchanan, John A. Stauffer, Horacio J. Asbun

**Affiliations:** ^1^Division of Gastroenterology and Hepatology, Mayo Clinic, 4500 San Pablo Road, Jacksonville, FL 32224, USA; ^2^Clinical Studies Unit, Mayo Clinic, 4500 San Pablo Road, Jacksonville, FL 32224, USA; ^3^Division of General Surgery, Mayo Clinic, 4500 San Pablo Road, Jacksonville, FL 32224, USA

## Abstract

*Objectives*. Surgery is the most effective treatment for pancreatic cancer. However, present literature varies on outcomes of curative pancreatic resection in the elderly. The objective of the study was to evaluate age as an independent risk factor for 90-day mortality and complications after pancreatic resection.* Methods*. Nine hundred twenty-nine consecutive patients underwent 934 pancreatic resections between March 1995 and July 2014 in a tertiary care center. Primary analyses focused on outcomes in terms of 90-day mortality and postoperative complications after pancreatic resection in these two age groups.* Results*. Even though patients aged 75 years or older had significantly more postoperative morbidities compared with the younger patient group, the age group was not associated with increased risk of 90-day mortality after pancreatic resection.* Discussion*. The study suggests that age alone should not preclude patients from undergoing curative pancreatic resection.

## 1. Introduction

The older population, defined as older than 65 years of age, is an important section of the United States (US) population [1]. The US 2010 census reported that the older population increased at a faster rate (15.7%) than the entire US population (9.7%) in the last decade [1]. This expanding older population has also been reported to have a higher incidence of pancreatic cancer. From 2008–2012, 39.6% of pancreatic cancers were diagnosed in patients older than 75 years of age [2]. The Surveillance, Epidemiology, and End Results program estimated that, in 2015, there were 48,960 new cases of pancreatic cancer and 40,560 people died of this disease [2]. Pancreatic cancer is the third leading cause of cancer death in the United States [2]. The 5-year survival rate for pancreatic cancer is 7.2% [2]. To date, surgery has proven to be the most effective treatment for pancreatic cancer. Despite the recent advances that have reduced the overall operative risks [3–6], there is still conflicting evidence regarding the outcomes of surgery in older patients.

The majority of the studies published on the topic have been retrospective, with small sample sizes, conducted at highly specialized, high-volume centers [4, 7–12]. Some studies have reported age to be a risk factor in postoperative morbidity after pancreatic resection [6, 11, 13–16], while others reported that increased age alone does not prohibit pancreatic surgery [7, 10, 12, 17]. Recently, Lee et al. reported that increased age is not a risk factor for postoperative mortality based on information from the American College of Surgeons National Surgical Quality Improvement Program (NSQIP) database [3]. However, NSQIP is a voluntary program, and it does not represent all hospitals in the US. Furthermore, the NSQIP database is built on a sample from a particular patient population, so it does not include the total number of patients treated at each participating institution. It is, therefore, difficult to correctly make a conclusion based on the current available findings.

The aim of this study was to compare the outcomes of pancreatic surgery, including 90-day morbidity and mortality, for patients aged 75 years or older to those younger than 75 years in a high-volume tertiary center.

## 2. Methods

A retrospective review of a prospectively maintained pancreas database was performed. All patients who had undergone pancreatic resection at our tertiary care center from March 1995 to July 2014 were included in the study. Institutional Review Board approval was obtained. All pancreatectomies were performed by the surgical group at our institution within the time period. This resulted in a sample of 934 pancreatic resections in 929 different patients. To optimize clinical outcome after pancreatic resection, preoperative decisions were preceded by individualized evaluations for the entire cohort. Every patient's cardiologist and/or pulmonologist for those with a medical history of cardiac or pulmonary disease, respectively, were consulted prior to the procedure for optimization of medical conditions including hypertension and diabetes. Ultimately, the decision to offer surgery was made by the attending surgeon after reviewing subspecialist evaluations and preoperative testing results and by assessing each patient's functional status via evaluation of an individual's capacity to perform activities of daily living independently. Our primary analysis focused on comparing morbidity and 90-day mortality of older patients undergoing pancreatic surgery to those younger than 75 years of age.

### 2.1. Data Collection and Outcomes

For baseline characteristics, age, gender, race, American Society of Anesthesiologists class, comorbidities, preoperative symptoms, and preoperative laboratory values were compared. For surgical details, type of resection, operative time, technique of pancreaticojejunostomy, gastrojejunostomy, and/or duodenojejunostomy, and pathology of the resected lesion were compared. For outcomes data, postoperative stay, including Intensive Care Unit (ICU) days, perioperative packed red blood cell transfusion, postoperative complications, and lab values; follow-up information; and postoperative mortality were compared. The primary outcome measures were to compare morbidities and 90-day mortality in the postoperative period between patients aged 75 years or older and those younger than 75 years.

### 2.2. Statistical Analysis

Continuous variables were summarized using the sample median with range and numbers with percentage. Comparison was done using *t*-test for continuous variables and chi-square test for categorical variables. Additionally, multivariate stepwise logistic regression was performed to estimate odds ratios for factors associated with 90-day mortality and the occurrence of major complications. *p* values less than 0.05 were considered significant. All statistical analyses were performed using SPSS for Windows version 17 (SPSS, Inc., Chicago, IL). This was not a funded study.

## 3. Results and Discussion

### 3.1. Results

The study sample included 934 pancreas resections: 737 aged younger than 75 years (range 18 years to 75 years) and 197 aged 75 years or older (range 75 years to 90 years of age). Patient demographics and comorbidities are shown in Table 1. Patients aged 75 years or older at the time of surgery were more likely to have a history of cardiovascular and pulmonary comorbidities and higher Eastern Cooperative Oncology Group (ECOG) status (Table 1). Active tobacco use was significantly lower in patients aged 75 years or older when compared to patients younger than 75 years (6% versus 19%). Patients younger than 75 years, as expected, were likely to have better health status as indicated by higher levels of hemoglobin (13.04 versus 12.51, *p* < 0.001), lower elevations of aspartate aminotransferase (50.61 versus 70.61, *p* = 0.003) and alkaline phosphatase (203 versus 283, *p* < 0.001), higher levels of albumin (4.04 versus 3.88, *p* < 0.001), and better renal functions (glomerular filtration rate 65.71 versus 61.18, *p* = 0.007). Overall, patients aged 75 years or older had higher morbidities than the patients younger than 75 years at the time of surgery.

Pathology of the resected lesions is shown in Table 1. Patients aged 75 years or older had a significantly higher incidence of pancreatic ductal adenocarcinoma (42.6% versus 31%) as compared to patients younger than 75 years. Surgical details of the resected specimen are shown in Table 2. Over the period of 20 years, there was a statistically significant trend towards pylorus preserving pancreaticoduodenectomy as compared to standard PD (*p* value <0.001). Patients aged 75 years or older were more likely to have pylorus preserving pancreaticoduodenectomy (PD) (32.9% versus 29.5%) and standard PD (22.3% versus 19.8%). There was no significant difference between the two groups in terms of operating time as shown in Table 2. The frequency of patients aged 75 years or older requiring packed red blood cell transfusion was significantly higher than those younger than 75 years (48% versus 35%, *p* < 0.0009).

A comparison of postoperative morbidity and mortality is shown in Table 1. Patients aged 75 years or older and those younger than 75 years had similar postoperative laboratory values except for the levels of INR (1.56 versus 1.80; *p* < 0.005). There were a total of 45 deaths within the 90-day postoperative period, for an overall mortality of 4.8%. The 90-day mortality rate for patients younger than 75 years was 3.2% (31), compared with 5% (14) for patients aged 75 years or older (*p* = 0.09). On multivariate analysis of preoperative factors associated with mortality (Table 3), age of 75 years or older was not associated with an increase in the likelihood of 90-day mortality (odds ratio 1.46; 95% confidence interval 0.74–2.87; *p* = 0.272). Despite no significant difference in 90-day mortality, postoperative complications were more significantly seen in patients aged 75 years or older as compared to patients younger than 75 years. A significantly higher proportion of patients aged 75 years or older experienced cardiac complications (17% versus 9%, *p* = 0.0001), pulmonary complications (15% versus 11%, *p* = 0.027), respiratory failure (8.5% versus 5%, *p* = 0.013), and renal insufficiency (5.5% versus 5%, *p* = 0.017).

### 3.2. Discussion

As the older population increases, there is an increasing demand for surgery in this older population. Outcomes of various surgical procedures, including PD, in the older population, have become a subject of concern; however, limited data exist [4, 7–12, 17]. It has been well established in previous studies that PD could safely be performed for patients aged 70 years or older. However, PD becomes daunting with patients who are older than 80 years. Outcomes in octogenarians have been reported over the last few years. While some of these studies have reported age as a risk factor in postoperative morbidity after PD [6, 11, 13–16], others have shown that age does not prohibit surgery [7, 10, 12, 17, 18]. One of the most recent studies on the topic, based on the NSQIP database [3], specifically analyzed outcomes of patients older than 80 years for increased risk of complications and mortality. Even though the study had a large sample size, participation in the NSQIP database is voluntary, so it does not represent all of the patients in the participating institutions, and not all hospitals in the US participate. Therefore, whether age plays a role in postoperative morbidity after PD or not remains a subject of further research.

This study has a large sample size and focuses on the population aged 75 years or older undergoing pancreatic resections at our tertiary center. We evaluated postpancreatic resection outcomes for patients aged 75 years and older as inconclusive evidence has been reported for octogenarians while good outcomes for septuagenarians have been well reported [7]. The results of the study are comparable to other studies reported on the topic [7, 10, 12, 17]. Overall mortality rate was 4.5%, closer to the mean of the 0–10% reported by the majority of the studies since 2000 [3, 19–22]. The incidence of postoperative fistula and hemorrhage is also comparable to previous studies [22–24]. Even though patients aged 75 years or older were more likely to have postoperative morbidities than their younger counterparts, 90-day mortality after pancreatic resection did not differ significantly. A higher incidence of postoperative complications in patients aged 75 years or older could also be explained by the higher incidence of preoperative morbidities in the same cohort as compared to the younger group. Comparable 90-day mortality could be reflective of the optimization of medical conditions prior to the procedure, low severity of postoperative complications in the older group, better postoperative care, or selection bias in terms of better health status prior to surgery. Despite the above, these findings suggest that age alone should not preclude patients from undergoing curative resection of pancreatic lesion.

This study is limited by its retrospective nature. By design, it spans over a time period of 20 years. Improved surgical techniques and advancements in perioperative care [4–6] have resulted in the surgeons offering PD to older patients at our tertiary center over the last 2 decades, and patients treated more recently may be doing better. Although it is well known that poor health status is associated with an increased risk of death and adverse outcomes [25, 26], this study cannot rule out selection bias as patients with overall better health status would have been selected for surgery based on ECOG scores (the included patients in the sample have ECOG scores between 0 and 2). In this study, occurrence of pancreatic ductal carcinoma (42.6% versus 31%) and solid pancreatic lesions (62.8% versus 54%) was more common in patients aged 75 years or older as compared to patients younger than 75 years, which could have influenced the outcomes as fibrotic pancreatic tissue is comparatively easier to resect. Lastly, even though the study has a large sample size, it is still underpowered for detecting minor differences between the two groups. It is also underpowered to determine preoperative risk factors in the older subgroup who have an increased number of ICU days or higher frequency of postoperative complications.

## 4. Conclusions

Focusing on the high-risk group of patients aged 75 years or older, this study reports that there is no significant difference in 90-day mortality despite higher postoperative complications in the subgroup. This study suggests that pancreatic resection could be a feasible option in selected older patients, and these patients should not be denied the opportunity of curative resection based on age alone.

## Figures and Tables

**Table 1:** Demographic details and postoperative morbidity.

Characteristic	Age < 75 years (*n* = 737)	Age ≥ 75 years (*n* = 197)	*p* value
Females (%)	54% (399)	49.2% (98)	0.26
Race			0.14
Caucasian	89% (655)	94.4% (188)	
Non-Caucasian	11% (82)	5.6% (9)	
Cardiovascular history			
Hypertension	56% (413)	66.8% (133)	0.001
Cardiac disease	40% (294)	76% (151)	0.001
Peripheral vascular disease	3.6% (27)	6% (13)	0.069
Pulmonary disease	8% (59)	11% (21)	0.23
Tobacco use			0.001
Past	41.5% (307)	53% (106)	
Active	19% (140)	6% (12)	
ECOG status			0.001
0	68.6% (517)	51.5% (103)	
1	27.7% (205)	40.5% (81)	
2	2.3% (17)	6% (12)	

Pathology			

Pancreatic ductal adenocarcinoma	31% (229)	42.6% (84)	0.002
Pancreatic cysts (IPMN, MCN, SCN)	21.9% (162)	20.3% (40)	0.61
Ampullary adenocarcinoma	6.5% (48)	11.1% (22)	0.03
Miscellaneous neoplasm (GIST, RCC, sarcoma, etc.)	2.5% (67)	8% (15)	0.51
Neuroendocrine tumors	13.7% (102)	5.5% (11)	0.002
Cholangiocarcinoma	8.5% (62)	9% (18)	0.74
Benign (pseudocyst, pancreatitis, trauma)	8.5% (67)	3.5% (7)	0.013

Postoperative morbidity and mortality			

90-day mortality	3.2% (31)	5% (14)	0.09
Mean number of ICU days	1.35 days	2.33 days	0.027
(Range)	(0–59 days)	(0–58 days)	
Clavien grade of complications (90 days)			0.56
(a) Minor (grades 1-2)	31% (227)	37% (73)	
(b) Major (grades 3–5)	21% (157)	22% (78)	
Pancreatic fistula	14.6% (108)	12% (24)	0.123
Grade A	6.3% (47)	5% (10)	
Grade B	4.2% (31)	4.5% (9)	
Grade C	4.2% (31)	2.5% (5)	
Postpancreatectomy hemorrhage	5% (38)	2.5% (5)	0.013
Grade A	1.2% (9)	0% (0)	
Grade B	1.2% (9)	1.5% (3)	
Grade C	2.7% (20)	1% (2)	

ECOG status: Eastern Cooperative Oncology Group (ECOG) performance status; IPMN: intraductal papillary mucinous neoplasm; SCN: serous cystic neoplasm; MCN: mucinous cystic neoplasms; GIST: gastrointestinal stromal tumors; RCC: renal cell cancer.

**Table 2:** Operative details.

Variable	Age < 75 years (*n* = 737)	Age ≥ 75 years of age (*n* = 197)	*p* value
Operative procedure			0.39
(a) Pancreaticoduodenectomy	49.5% (365)	55.3% (109)	
(b) Total pancreatectomy	10% (74)	12.6% (25)	
(c) Distal pancreatectomy	37% (274)	30.9% (61)	
(d) Central Pancreatectomy/enucleation	3% (24)	1% (2)	
Operative time (minutes)	339	348	0.40
Number of patients requiring blood transfusion perioperative^*∗*^	35% (260)	48% (95)	0.0009
Mean number of packed RBC transfused	1.42 units	1.75 units	0.32
Number of patients requiring blood transfusion during hospital stay in postoperative period	26% (197)	32% (64)	0.11
Mean number of packed RBC transfused	0.96 units	0.83	0.64

^*∗*^up to 24 hours after surgery. RBC: red blood cells.

**Table 3:** Multivariate logistic regression analysis for preoperative factors associated with mortality.

Variable	Odds ratio (95% confidence interval)	*p* value
Age of 75 years or older	1.46 (0.74–2.87)	0.272
Hypertension	0.501 (0.228–1.09)	0.084
Diabetes mellitus	0.63 (0.33–1.20)	0.161
Coronary artery disease	0.479 (0.239–0.960)	0.038
Pulmonary disease	0.449 (0.196–1.02)	0.058
